# An oil-absorbing resin with a simple polymerization system with benzyl methacrylate as a functional monomer

**DOI:** 10.1098/rsos.230343

**Published:** 2023-10-11

**Authors:** Yanjia Zhou, Ming Zhang, Yongqiang Liu, Bo Xu, Haiyan Qiu, Xiuli Che, Guihong Lan

**Affiliations:** ^1^ Department of Chemistry and Chemical Engineering, Southwest Petroleum University, Chengdu 610500, People's Republic of China; ^2^ China Railway Water Group Co., Ltd., Xi'an 710100, People's Republic of China; ^3^ China Tiegong Investment & Construction Group Co., Ltd., Beijing 100000, People's Republic of China; ^4^ Faculty of Engineering and Physical Sciences, University of Southampton, Southampton, UK

**Keywords:** oil-absorbing resin, oil absorbency, copolymerization

## Abstract

To solve the problem of the low absorbency of oil-absorbing resins, oil-absorbing resins (PAMs) were fabricated in this study by introducing commercially available benzyl methacrylate (BZMA) as a functional monomer copolymerized with stearyl methacrylate (SMA) and butyl acrylate (BA). The internal network structure of the PAMs expanded more easily when absorbing oils or organic solvents after introducing rigid groups of the benzene ring by an uncomplex polymerization process, which provided the oil-absorbing resin with good absorbency. The reagents were all commercially available, and there was no other pretreatment or posttreatment process. Then, the optimum parameters for the monomer feed ratio, water/oil mass ratio, and concentrations of initiator, stabilizer and crosslinker were studied. Simultaneously, the reusability, oil retention and thermal stability of PAMs were investigated in this article. The PAMs swelled in various oils and organic solvents (the values of oil absorbency were 44.52, 56.13, 25.54, 28.21, 32.85, 24.56, 14.17, 15.02 and 29.07 g g^−1^ for CCl_4_, CHCl_3_, CH_2_Cl_2_, benzene, toluene, xylene, n-hexane, 0# diesel oil and 93# gasoline, respectively) and displayed good oil absorbency, which met the absorption requirements for common oils or organic solvents.

## 1. Introduction

Oil-absorbing resins, as ideal oil-absorbent materials, are extensively used for oily wastewater remediation, and display high oil absorbency and good oil/water selectivity, oil retention and reusability [1]. Due to these superb properties, oil-absorbing resins have vital practical influence and bright prospects for use in oily wastewater treatment, which is attributed to the fact that an oil-absorbing resin is a polymer with a low crosslinked three-dimensional network [2]. The structure of oil-absorbing resin can ensure swelling without dissolution of the resin in oils and organic solvents to achieve the purpose of oil and organic solvent adsorption [3].

In fact, the performance of oil-absorbing resin before and after oil absorption can be adjusted by adjusting the composition of the polymerization system. The use of copolymerized monomers is extremely significant for material capabilities [4]. Thus, it is necessary to adopt various compositions of monomers to create a novel oil-absorbing resin. When selecting monomers to prepare oil-absorbing resins, (meth)acrylate from various sources is the main focus and has advanced polymerization technology associated with it [5].

Endeavours have been made in the studies of polymerization systems of oil-absorbing resins, such as the study by Ran *et al*. [6] in which an oil-absorbing resin was synthesized with butyl acrylate (BA) and methyl methacrylate (MMA) as monomers. This resin had the advantage of using a readily available and low-cost raw material, but its oil absorbency was low (26.84 g g^−1^ for CHCl_3_ and 14.15 g g^−1^ for toluene).

To overcome the defect of low oil absorbency, a series of studies were conducted, such as introducing monomers with long side chains. Sun *et al*. [7] chose lauryl methacrylate (LMA), hexadecyl methacrylate (HMA) and stearyl methacrylate (SMA) as monomers to polymerize three oil-absorbing resins; the oil absorbency improved to 24, 32 and 34 g g^−1^ for CHCl_3_, respectively. Geng *et al*. [8] designed an oil-absorbing resin using SMA, BA and styrene (St) as monomers, and the oil absorbency reached 34.00 and 23.18 g g^−1^ for CHCl_3_ and toluene, respectively. Despite the improvement in oil absorbency in the above studies, there is still room for advancement.

Ding *et al*. [9] prepared an oil-absorbing resin by compounding a novel functional monomer consisting of β-cyclodextrin with a torus-shaped ring structure. The prepared resin in this paper had a great oil absorbency (75 and 55 g g^−1^ for CHCl_3_ and toluene, respectively). Unfortunately, there are certain disadvantages: synthesizing an innovative monomer involves processes such as preparation, purification and characterization, which are complicated, time-consuming, costly and difficult to mass produce.

Hence, an effective way to improve the applicability of oil-absorbing resin is to prepare resins with good oil absorbency by a simple synthesis system and polymerization process.

To boost oil absorbency, in this study benzyl methacrylate (BZMA) with benzene ring groups was introduced as a functional monomer, which can contribute to the formation of a structure that both has a rigid backbone and is easily extended. It is predicted that BZMA, as a functional monomer of copolymerization, is qualified to provide more space volume, enhance mechanical strength and decrease the entanglement of long-chain alkyl groups to stretch easily [10].

This study presents a new oil-absorbing resin (PAM) and delves into how the properties of oil-absorbing resin are influenced by the monomer composition in the polymerization system. Furthermore, optimization of other parameters (the monomer ratio, water/oil mass ratio, and initiator, crosslinker and stabilizer concentration) is investigated. This provides further insight into developing and designing new oil-absorbing resins. Without the intricate preparation of new functional monomers and additional pretreatment or posttreatment processes, the oil-absorbing resin achieved good oil absorbency by introducing commercially available reagents to the synthesis system directly. Since the produced PAMs show somewhat good performances, they are realistically expected to be applied for oily water treatment.

## 2. Experimentation

### 2.1. Materials

SMA (96%) and BZMA (98%) were obtained from Aladdin Chemistry Co., Ltd. Butyl acrylate (BA, AR), dibenzoyl peroxide (BPO, AR), N,N'-methylenebis (2-propenamide) (MBA, AR), toluene, xylene, acetone and n-hexane were obtained from Chengdu Kelon Chemical Reagent Factory. Polyvinyl alcohol (PVA, CP) was obtained from Shanghai Chemical Laboratory Equipment Co., Ltd. Tetrachloromethane was obtained from Shanghai Maclean Biochemical Technology Co., Ltd. Chloroform, dichloromethane, benzene and ethyl alcohol were obtained from Chengdu Cologne Chemicals Co., Ltd. All reagents above were used as received.

### 2.2. Preparation of PAMs

First, a given weight of stabilizer (PVA) was completely dissolved in deionized water by utilizing a 250 ml three-neck flask with a stirrer, reflux condenser and gas inlet pipe. Polymerizations were carried out in a water bath, and the system temperature was slowly increased to 80°C. A mixture of monomers (SMA, BA and BZMA) with initiator (BPO) and crosslinker (MBA) was added to the PVA aqueous solution. After 6 h of suspension polymerization at a stirring speed of 200 rpm under a N_2_ atmosphere, the prepared oil-absorbing resin was collected by filtration when the product cooled to ambient temperature. Then, the resin was washed three times with deionized water and ethanol. Following this treatment, the prepared resin was dried in a vacuum oven at 40°C until reaching a constant weight. Finally, a series of PAMs were synthesized [11,12]. All the ingredients used are summarized in table 1.

**Table 1.** Materials in the PAMs polymerization system.

material	dosage(g)
SMA	7.14
BA	1.43
BZMA	1.43
H_2_O	50.00
PVA	0.05
MBA	0.05
BPO	0.05

### 2.3. Oil absorption test

To quantify the performance of the oil-absorbing resin, the following parameters were investigated: oil absorbency, oil absorption speed, swelling kinetics, oil retention percentage, reusability and crosslinking degree. All measurements were performed in triplicate, and the value of the oil absorbancy was the average of three results from repeated tests.

### 2.4. Oil absorbency of PAMs

The oil absorbency testing approach was based on ASTM F726-81 [13], and the oil absorbency was calculated as the ratio of oil adsorbed to dry resin weight by equation (2.1).

2.1 $$\begin{array}{c} \mathrm{Oil}\;\mathrm{absorbenc}y_{m}=\frac{m_{T}-m_{0}}{m_{0}}, \end{array}$$

where *m*_T_ is the weight of the PAMs sample at the end of the oil tests, and *m*_0_ is the initial dry resin weight.

### 2.5. Oil absorption speed of PAMs

The oil absorption speed of PAMs was determined by weighing the swollen resin sample after an immersion interval of approximately 10–15 min, according to the measurement described in §2.4.

### 2.6. Swelling kinetics of PAMs

The swelling kinetics of oil absorption were studied by measurements in §2.5. For first-order sorption, the oils or organic solvents were absorbed, and the swelling rate was described by the equation (2.2) [14],

2.2 $$\begin{array}{c} \frac{dQ}{dt}\;=K_{1}(Q_{\max}-Q_{t}), \end{array}$$

where *Q*_max_ is the saturated absorbency of the PAMs, *Q_t_* is the oil absorbency of the PAMs at time *t* and *K*_1_ is the kinetic equilibrium rate constant of first-order sorption [15]. Equation (2.2) can be integrated into equation (2.3),

2.3 $$\begin{array}{c} -\log(Q_{\max}-Q_{t})=\frac{K_{1}}{2.303}t+C, \end{array}$$

where *t* is the sorption time, and *C* is the integration content. As *t* = 0, *Q_t_* = 0, and −log*Q*_max_ = C. Therefore,

2.4 $$\begin{array}{c} \log\frac{Q_{\max}}{Q_{\max}-Q_{t}}=\frac{K_{1}}{2.303}t \end{array}.$$

Plotting $\log(Q_{\max}/(Q_{\max}-Q_{t}))$ versus *t* yields a straight line with a slope of *K*_1_ [16].

For the second-order sorption kinetic model, the swelling rate was represented by the equation (2.5),

2.5 $$\begin{array}{c} \frac{dQ}{dt}\;=K_{2}{\left(Q_{\max}-Q_{t}\right)}^{2} \end{array}.$$

Equation (2.5) can be integrated into equation (2.6) for the integral interval 0 to *t* for *t* and 0 to *Q*_*t*_ for *Q*_*t*_, yielding

2.6 $$\begin{array}{c} Q_{t}=\frac{K_{2}Q_{\max}^{2}t}{1+K_{2}Q_{\max}t} \end{array}.$$

Rearranging equation (2.6) yields the following linear formula:

2.7 $$\begin{array}{c} \frac{Q_{t}}{Q_{\max}(Q_{\max}-Q_{t})}=K_{2}t, \end{array}$$

where *K*_2_ is the kinetic equilibrium rate content of second-order sorption [17]. The *K*_2_ was determined through the linear relation in equation (2.7).

### 2.7. Oil retention of PAMs

The tests referred to the method studied by Schott *et al*. [17] and the oil retention rate was calculated by equation (2.8),

2.8 $$\begin{array}{c} \mathrm{Oil}\;\mathrm{retention}\;\mathrm{rate}=\frac{m_{C}-m_{0}}{m_{S}-m_{0}}\times 100\text{\%}, \end{array}$$

where *m*_S_ is the weight of the fully swollen absorbent, *m*_C_ is the mass of the resin sample after centrifugation and *m*_0_ is the initial dry absorbent weight. 24 h is required for absolute oil absorption to reach the absorption saturation.

### 2.8. Reusability of PAMs

The saturated resins were desorbed in ethanol for 24 h and dried in a vacuum oven at 80°C until a constant weight was reached [18]. The reusability of the PAMs was investigated by repeating the procedure described in §2.7. Up to 10 cycles were carried out on a resin absorbent.

### 2.9. Crosslinking degree

The tests referred to the method studied by Sun *et al*. [7] and the crosslinking degree (D) was calculated by equation (2.9),

2.9 $$\begin{array}{c} D=\frac{m_{2}}{m_{1}}\times 100\text{\%} \end{array},$$

where *m*_1_ is the initial dry absorbent weight, and *m*_2_ is the mass of the resin sample at the end of oil absorption and drying.

### 2.10. Characterizations

The chemical molecular structures of PAMs were investigated by infrared (IR) spectroscopy. FT-IR spectra were collected using KBr pellets of samples on a WQF-520 FT-IR spectrometer. The surface morphologies of PAMs were observed by scanning electron microscopy (SEM) on a ZEISS Sigma 300 (Germany). The sample was directly glued to the conductive adhesive, and gold was sputtered for 45 s at 10 mA with a Quorum SC7620 sputtering coater. A Hitachi Regulus 8100 scanning electron microscope was then used to photograph the morphology of the sample. The acceleration voltage was 3 kV, and the detector was an SE2 secondary electron detector. Thermal stability was examined with thermogravimetric analysis on a Mettlaer TGA 2 (Switzerland) at a temperature range of 30–800°C and a heating rate of 10°C min in a N_2_ atmosphere. BET analysis was examined with Quantachrome-EVO at 120°C, and the qualitative masses of the samples were taken.

## 3. Results and discussion

### 3.1. Fabrication route for preparing PAMs

To reach higher oil absorbency, appropriate acrylic esters with different alkyl chain lengths were routinely used for copolymeriziton [19].

Oil absorbency is determined by the affinity between resin and oil in accordance with the theory of like-dissolves-like. Long-chain alkyl acrylate is characterized by hydrophobicity and commonly has a high affinity for oil and nonpolar solvents [20]. The longer the alkyl group of acrylates was, the higher the oil affinity and the lower the glass transformation temperature of the polymer [21]. Consequently, SMA served as the main monomer in the polymerization system. It enhances the oil-absorbing ability but weakens the mechanical strength of the polymer, which is not conducive to retaining and transporting oil after absorption [22].

In addition, SMA with long-chain alkyl groups experiences entanglement due to van der Waals forces, which results in partial physical crosslinks weaker than the chemical crosslinks formed to expand the efficient structure volume [23]. Nevertheless, excess physical crosslinks can make it difficult for long-chain alkyl groups to stretch during the oil absorption process. To overcome this defect and maintain its advantages, introducing BA could provide a partially effective space volume [24].

**Figure 1.**
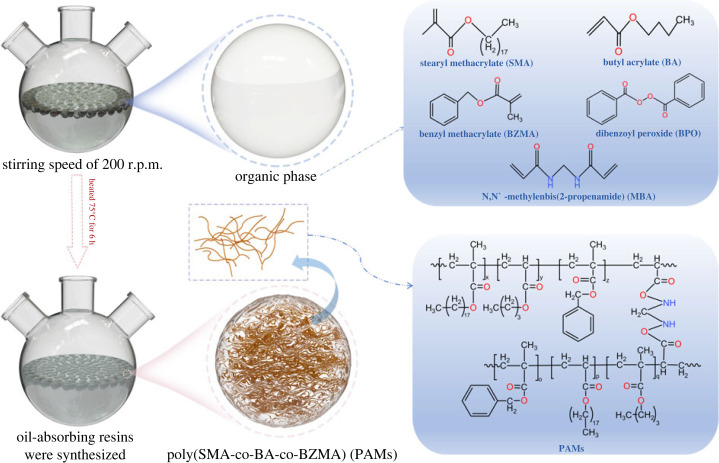
Fabrication route of PAMs.

P(SMA-co-BA-co-BZMA) oil-absorbing resins (PAMs) were prepared via suspension polymerization [25,26]. The as-prepared samples were synthesized following the fabrication route depicted in figure 1.

### 3.2. Characterization analysis of PAMs

**Figure 2.**
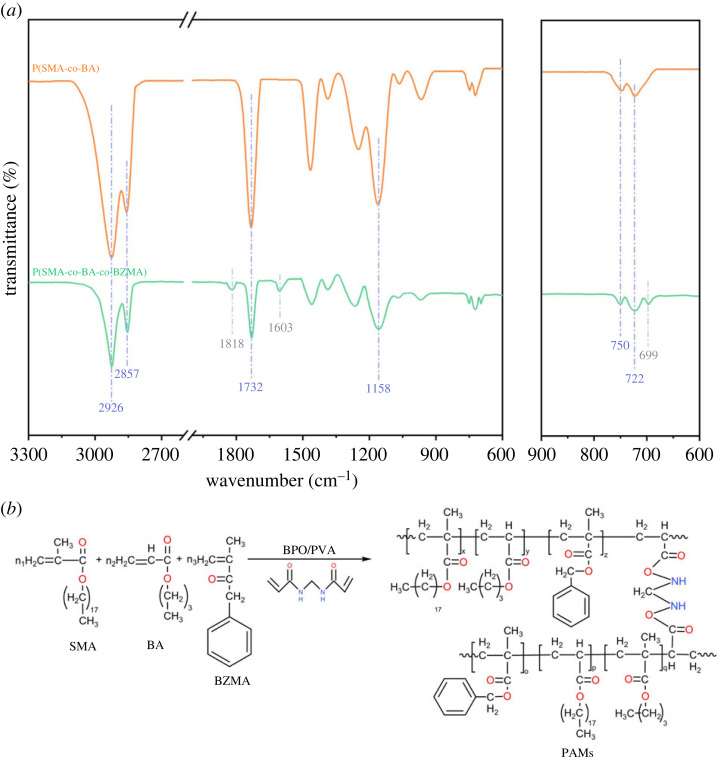
FT-IR spectra of P(SMA-co-BA) and P(SMA-co-BA-co-BZMA) (*a*), and the synthesis equation of PAMs (*b*).

The molecular structures of P(SMA-co-BA) and PAMs were examined by Fourier transform infrared spectroscopy, as shown in figure 2. The peaks at 2926 and 2857 cm^−1^ were attributed to C-H asymmetric and symmetric vibrations, respectively; the peaks at 1732 and 1158 cm^−1^ were attributed to C = O and C-O-C stretching vibrations, respectively; and the peaks at 722 and 750 cm^−1^ were attributed to -CH_2_ in-plane rocking vibrations in SMA and -CH_2_ rocking vibrations in BA and SMA, respectively. Moreover, the absorbance at 699 cm^−1^ corresponded to the out-plane flexural vibration of C-H in the benzene ring, and those at 1818 and 1603 cm^−1^ corresponded to the skeleton vibration of benzene, which appeared in PAMs only. These results reflected that BZMA, as a functional monomer, was successfully introduced into the copolymerization system and reacted with SMA and BA to synthesize the terpolymer PAMs.

**Figure 3.**
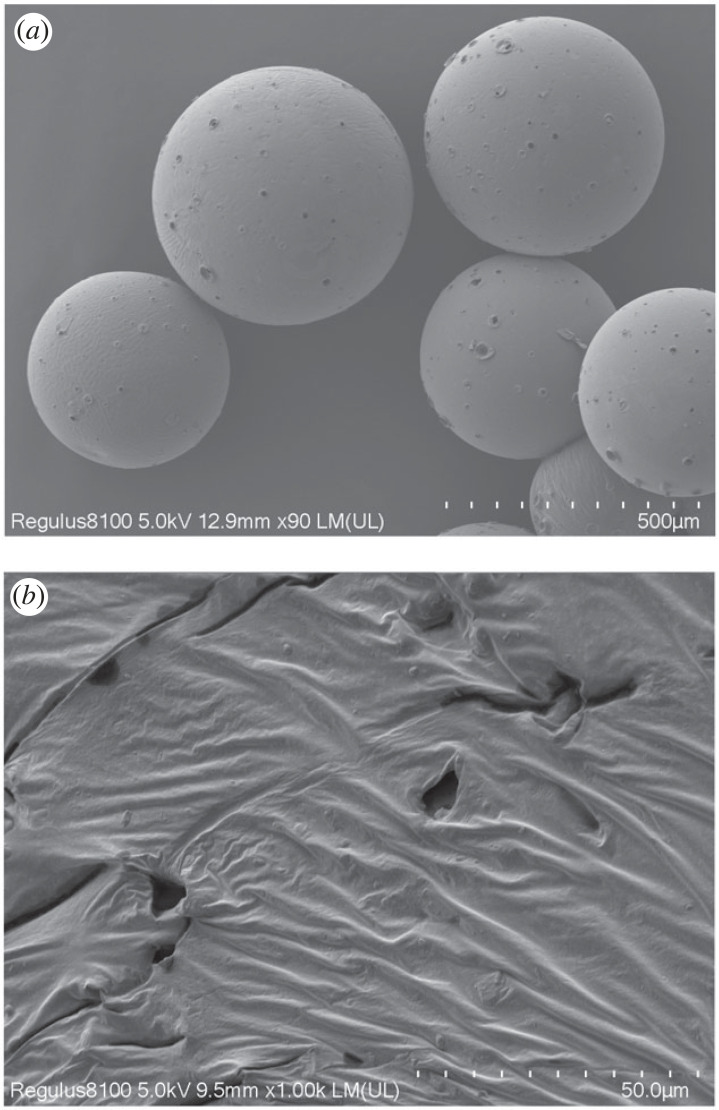
SEM images of the PAMs. (Magnification of (*a*) was 90 X and (*b*) was 1000 X.)

SEM images of PAMs with the gold-plating process are shown in figure 3. The photographs in figure 3*a*,*b* show the surface morphology of PAMs. It was apparent that the surface of PAMs was covered with uneven wrinkles, demonstrating that these wrinkles enlarged the specific surface area of PAMs, resulting in an increase in oil absorbency [27]. As seen from the figure, PAMs are spherical particles with a pore structure. A few holes were observed on the surface of the PAMs. It can be interpreted that the PAMs were terpolymers with a low crosslinking degree and formed a three-dimensional network by the copolymerization of several monomers, and there were certain internal pores [19,28].

### 3.3. Parameter optimization of synthetic system

CHCl_3_ and toluene, as typical organic contaminants of BTEX and halohydrocarbon, are among the most commonly used organic solvents and cause significant pollution if they enter water environments [29]. Thus, the properties of PAMs during the parameter optimization were tested with these two organic solvents as the objects to be absorbed.

### 3.4. Effects of BZMA dosage on oil absorbency

**Figure 4.**
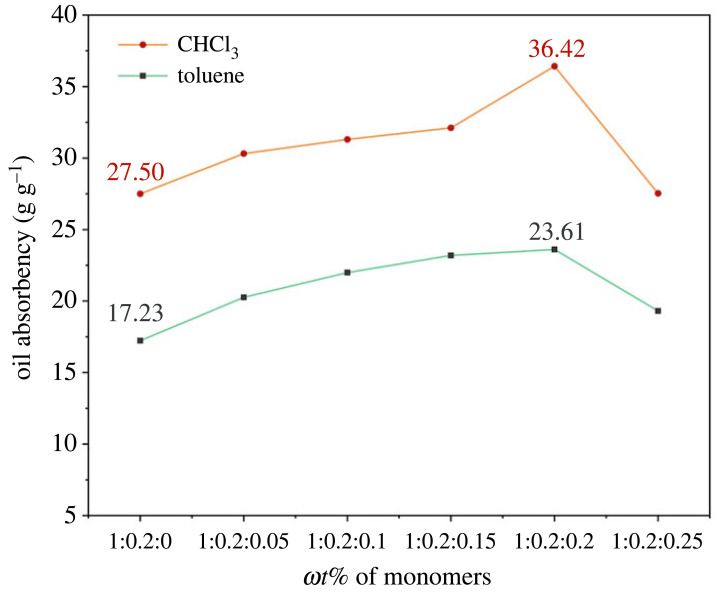
Effects of monomer ratio on the oil absorbency of the PAMs (*m*_water_/*m*_oil_ = 4 : 1 using 1 *ω*t% BPO as the initiator and 0.5 *ω*t% MBA as the crosslinker) in two oils.

The correlational results obtained from the preliminary analysis of the oil absorbency of PAMs are illustrated in figure 4. When the feed ratio of SMA and BA was *ω*(SMA : BA) = 1 : 0.2, the resin samples showed a maximum absorbency of up to 17.23 g g^−1^ for toluene and 27.50 g g^−1^ for CHCl_3_. The short chain of BA might reduce the entanglement and crystallization of longer alkyl chains of SMA, i.e. the physical crosslinked structure weakened and the effective volume supplying oil absorption expanded [30].

Apparently, oil absorbency increased after adding BZMA as a functional monomer. It can be speculated that the benzene ring, as a large and rigid group, occupied a certain network volume and supported the internal skeleton structure of the resin because of its steric effect [31].

However, the curve of oil absorbency declined with the continuous addition of BZMA after reaching a peak. The excess amount of BZMA quickly boosted the content of benzene rings between crosslinked points, which led to excessive rigidity so that the effective structure volume was occupied by large benzene rings [32]. On the other hand, it was difficult to cause swelling because of the surplus of hard monomers [33], so the oil absorbability decreased rapidly. Hence, the optimum monomer feed ratio was *ω*(SMA : BA : BZMA) = 1 : 0.2 : 0.2.

### 3.5. Effects of water/oil mass ratio and initiator dosage on oil absorbency

Water can serve as the dissolved and dispersed phase, which influences the transfer mass and heat in the suspension reaction. The initiator concentration has a significant impact on the length of the polymer chains of the crosslinked points [34,35].

**Figure 5.**
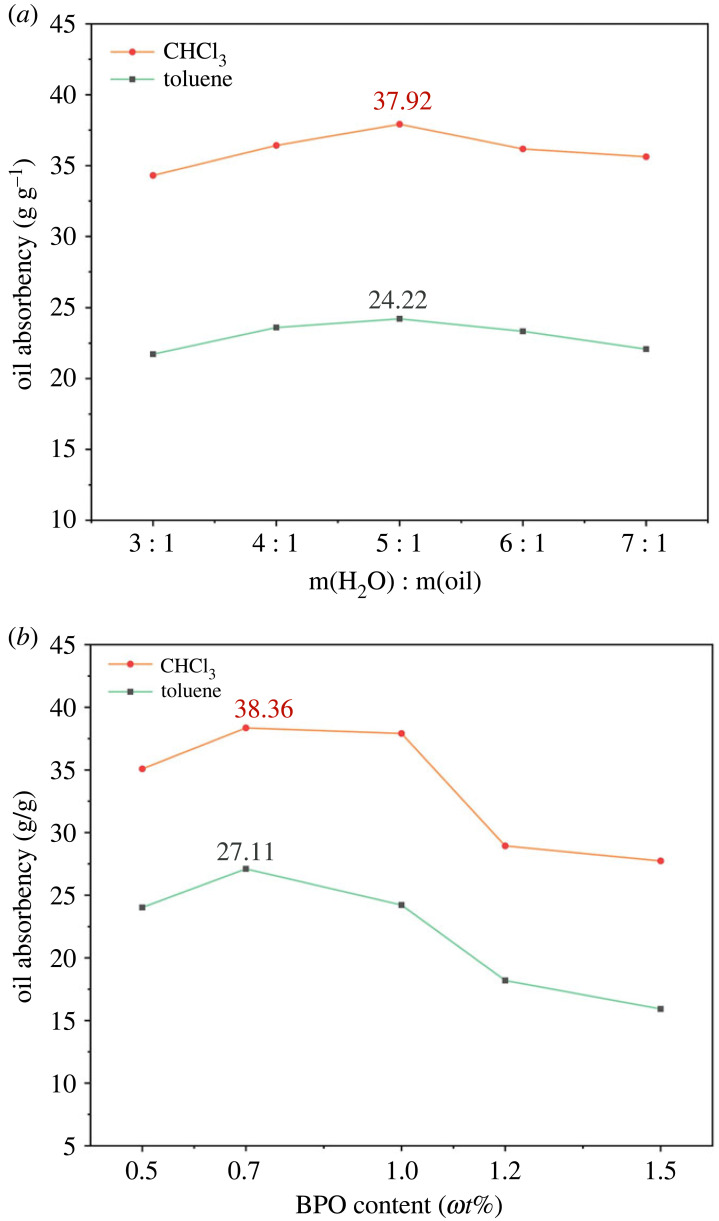
Effects of the water/oil mass ratio on the oil absorbency of the PAMs (*a*) (*ω*_SMA_/*ω*_BA_/*ω*_BZMA_ = 1 : 0.2 : 0.2 using 1 *ω*t% BPO as the initiator and 0.5 *ω*t% MBA as the crosslinker) in two oils. Effects of the BPO content on the oil absorbency of the PAMs (*b*) (*ω*_SMA_/*ω*_BA_/*ω*_BZMA_ = 1 : 0.2 : 0.2 and *m*_water_/*m*_oil_ = 5 : 1 using 0.5 *ω*t% MBA as the crosslinker) in two oils.

Figure 5 reveals the variation in the relationship between the water/oil mass ratio, initiator dosage and oil absorbency. These results suggest that oil absorbency was the highest when the water/oil mass ratio was 5 : 1, and the oil absorbency reached a maximum at a BPO content of 0.7 *ω*t%.

The lower water phase in the polymerization system led to a higher ratio of the oil phase, in which the heat of polymerization hardly dissipated. Then, the reaction rate accelerated, and the molecular weight of the chain segment between crosslinked points was small, while the oil absorption rate decreased. In addition, with the amount of water phase added, the heat dissipation of the polymerization reaction was fast, resulting in a slow reaction rate [36]. The average molecular weight of the chain segment between crosslinked points was large, and the soluble part increased while the degree of polymerization increased, whereas the oil absorption rate decreased [10].

The long-chain resin was synthesized at a low BPO concentration. There were few active centres because not enough monomers were activated and remained in the reaction system after a period. The loose crosslinked network was formed, resulting in a drop in oil absorbency [34]. By contrast, a higher BPO concentration brought about the formation of short polymeric chains, which was adverse in terms of oil absorbency [35].

### 3.6. Effect of stabilizer dosage on oil absorbency

The above suspension polymerization experiments were carried out without stabilizers using SMA, BA and BZMA as monomers, MBA as a crosslinker, and BPO as an initiator, and the shape of PAMs synthesized in this way was lumpy. Spheres have the largest volume per unit surface area, and the volume used to absorb and save oils was largest when the contact area between oil and resin was equal (the results of BET analysis of the equal mass PAMs prepared with no PVA and 1.0 *ω*t% PVA are shown in electronic supplementary material, figure S1*a*,*b*, respectively). The stabilizer decreases the interfacial tension between the oil phase and the water phase so that the oil phase sustains the state of oil droplets during the process of polymerization, and the resultant products are spherical. Thus, for polymerizing spherical oil-absorbing resins, PVA with great dispersion ability was added into the synthesized system and served as a stabilizer.

**Figure 6.**
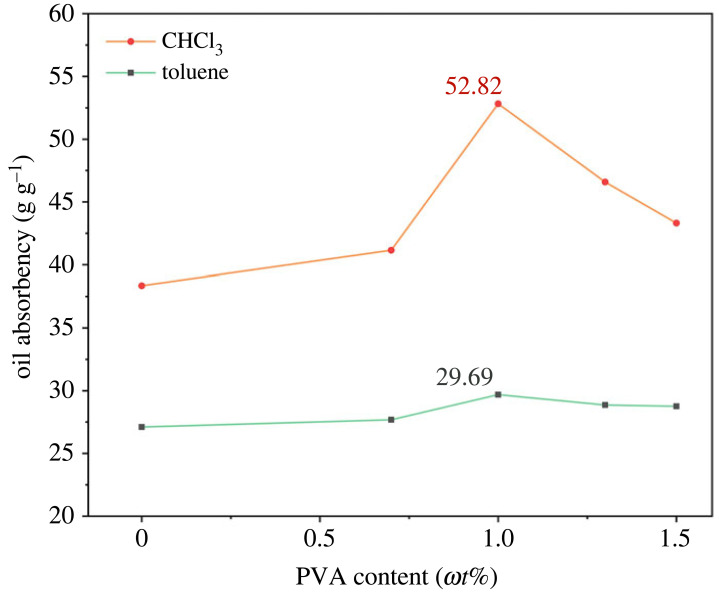
Effects of PVA content on the oil absorbency of the PAMs (*ω*_SMA_/*ω*_BA_/*ω*_BZMA_ = 1 : 0.2 : 0.2 and *m*_water_/*m*_oil_ = 5 : 1 using 0.7 *ω*t% BPO as initiator and 0.5 *ω*t% MBA as the crosslinker) in two oils.

In addition, the dosage of stabilizer has a substantial impact on the stability and size of particles and the heat transfer of the system. PVA as a stabilizer was added to the polymerization system with varied PVA contents (from 0.7 *ω*t% to 1.5 *ω*t%, based on the total weight of monomers) to analyze the interrelationship between stabilizer content and oil-absorbing performance. Figure 6 shows the relationship between stabilizer dosage and oil absorbency.

Obviously, the oil absorbency of PAMs synthesized by introducing PVA into the reaction system increased dramatically compared with those synthesized without a stabilizer. Simultaneously, the oil absorbency of PAMs rose and then declined after reaching a peak as the PVA dosage increased. The oil phase easily reagglomerated in the polymerization process at low PVA concentrations, resulting in less spherical and less uniform PAM products and ultimately leading to a reduction in oil absorbency (the SEM image of PAMs prepared with no PVA dosage is shown in electronic supplementary material, figure S2). Conversely, when the dosage of PVA was in excess, PVA remained on the surface of the PAMs after polymerization was completed. It was difficult to remove PVA in the posttreatment, which also caused a decrease in the oil absorbency [37]. Accordingly, the optimal dosage of PVA as a stabilizer was 1.0 *ω*t% in this polymerization system.

### 3.7. Effect of crosslinker concentration on oil absorbency

**Figure 7.**
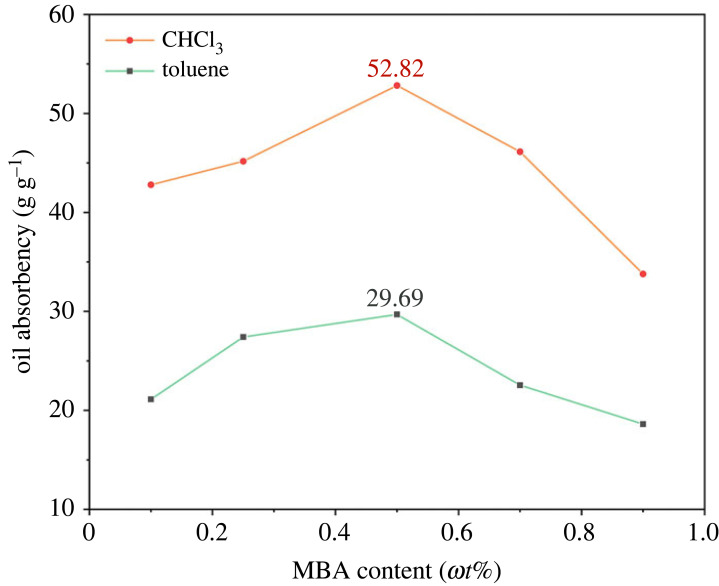
Effects of MBA content on the oil absorbency of the PAMs (*ω*SMA/*ω*BA/*ω*BZMA = 1 : 0.2 : 0.2 and *m*_water_/*m*_oil_ = 5 : 1 using 0.7 *ω*t% BPO as the initiator and 1.0 *ω*t% MBA as the stabilizer) in two oils.

The type and concentration of crosslinker have a crucial effect on the properties of the oil-absorbing resin. The copolymerization of monomers and a crosslinker can lead to resin synthesis in a three-dimensional network space structure. A series of PAMs were prepared with varied concentrations of MBA (from 0.1 *ω*t% to 0.9 *ω*t%) to evaluate oil absorbency to study the effect of the crosslinker. Figure 7 shows that the oil absorbency first increased and then decreased with an increasing MBA amount, and the optimal concentration of MBA as a crosslinker was 0.5 *ω*t%.

When the concentration of crosslinker was low, the crosslinking degree of PAMs was small, so that the polymeric products contained certain soluble parts [38], forming an invalid crosslinking, which made the resin unable to maintain an efficient crosslinked network structure. Thus, the poor mechanical properties after oil adsorption usually strongly held PAMs in their spherical shape and even made them dissolve in oil. However, the PAMs formed a dense crosslinked network when the amount of MBA was large. It might be interpreted that the chain length between crosslinked points was reduced, and it was difficult for the side chains of polymers to expand [23], which meant that the effective volume occupied by oil or organic solvent was limited. The above reasons might cause the oil absorbency of the PAMs to decrease.

### 3.8. The orthogonal experiment of PAMs

The synthesis conditions were further optimized by an orthogonal experiment, and the orthogonal experiment was carried out by selecting the four-factor L9 (3^4^) orthogonal table. The orthogonal experimental scheme and results are shown in table 2. It was shown that the PAMs combination with the best CHCl_3_ and toluene adsorption capacity was: A_2_B_3_C_3_D_2_.

**Table 2.** PAMs orthogonal experiment design and results.

factors' influence and level		A		B	C	D	
		*ω*(SMA : BA : BZMA)		*ω*(BPO)/%	*ω*(MBA)/%	*ω*(PVA)/%	
1		1 : 0.2 : 0.15		1.0 *ω*t%	0.4 *ω*t%	1.0 *ω*t%	
2		1 : 0.2 : 0.2		0.7 *ω*t%	0.6 *ω*t%	0.5 *ω*t%	
3		1 : 0.2 : 0.25		0.5 *ω*t%	0.5 *ω*t%	1.5 *ω*t%	
experiment number		A	B	C	D	toluene	CHCl_3_
1		1	1	1	1	20.09	38.15
2		1	2	2	2	20.56	38.25
3		1	3	3	3	27.00	45.08
4		2	1	2	3	28.18	50.91
5		2	2	3	1	24.37	46.67
6		2	3	1	2	31.61	55.40
7		3	1	3	2	23.38	48.48
8		3	2	1	3	19.19	42.90
9		3	3	2	1	22.69	45.08
toluene	K_1_	67.65	71.65	70.89	67.15	/	
	K_2_	84.16	64.12	71.43	75.55		
	K_3_	65.26	81.30	74.75	74.37		
	k_1_	22.55	23.88	23.63	22.38		
	k_2_	28.05	21.37	23.81	25.18		
	k_3_	21.75	27.10	24.92	24.79		
	R	6.30	5.73	1.29	2.80		
CHCl_3_	K_1_	121.48	137.54	136.45	129.90	/	
	K_2_	152.95	127.82	134.24	142.13		
	K_3_	136.46	145.56	140.23	138.89		
	k_1_	40.49	45.85	45.48	43.30		
	k_2_	50.98	42.61	44.75	47.38		
	k_3_	45.49	48.52	46.74	46.30		
	R	10.49	5.91	1.99	4.08		

### 3.9. Oil absorbency on common oils and organic solvents

Preparing PAMs with the above-described optimal parameters (*ω*(SMA : BA : BZMA) = 1 : 0.2 : 0.2, *ω*(BPO) = 0.5 *ω*t%, *ω*(MBA) = 0.5 *ω*t% and *ω*(PVA) = 0.5 *ω*t%) yielded an oil-absorbing resin with a great absorbency of up to 32.85 g g^−1^ for toluene and 56.13 g g^−1^ for CHCl_3_. The values of oil absorbency of PAMs for toluene and CHCl_3_ were compared with other oil-absorbing resins that used acrylate monomers (table 3).

**Table 3.** Comparison of oil absorbency.

oil-absorbing resin	oil absorbency/g·g^−1^		ref.
	toluene	CHCl_3_	
poly (BA-co-MMA)	14.15	26.84	[6]
poly(BMA-co-BA)	16.72	36.51	[39]
poly (SMA-co-BA-co-St)	23.18	34.00	[8]
poly(ODA-co-BA)	31.8	41.8	[40]
^a^poly (SA-co-St-co-β-CD-MA)	55.00	75.00	[9]
PAMs	32.85	56.13	this paper

**Note: (a)** The functional monomer *β*-CD-MA was compounded with *β*-cyclodextrin.

Apparently, the oil absorbency of PAMs was higher than that of the oil-absorbing resins synthesized with a simple polymerization system. And the oil absorbency of PAMs was lower than that of oil-absorbing resin modified by β-cyclodextrin, but β-cyclodextrin is expensive and its modification process is complicated and time-consuming. Obviously, PAMs without intricate treatment processes had a satisfactory oil-absorbing performance in a simple synthesizing process.

**Figure 8.**
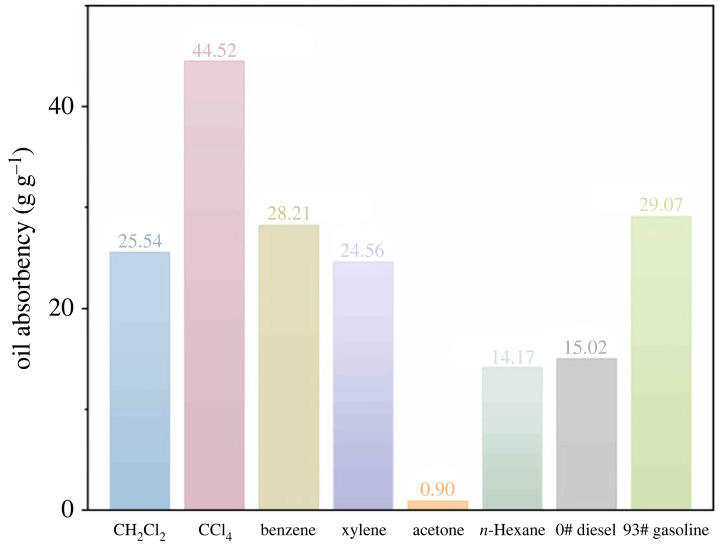
Oil absorbency of PAMs on various oils and organic solvents.

Furthermore, to evaluate the all-side oil-absorbing performance of PAMs, resins were immersed in other oils and organic solvents (CH_2_Cl_2_, CCl_4_, benzene, xylene acetone, n-hexane, 0# diesel oil and 93# gasoline). In addition to acetone, certain oil-absorbing properties were exhibited by PAMs on various oils and organic solvents. Figure 8 shows that PAMs have potential practical applications in oily wastewater abatement.

### 3.10. Swelling kinetic analysis of PAMs

**Figure 9.**
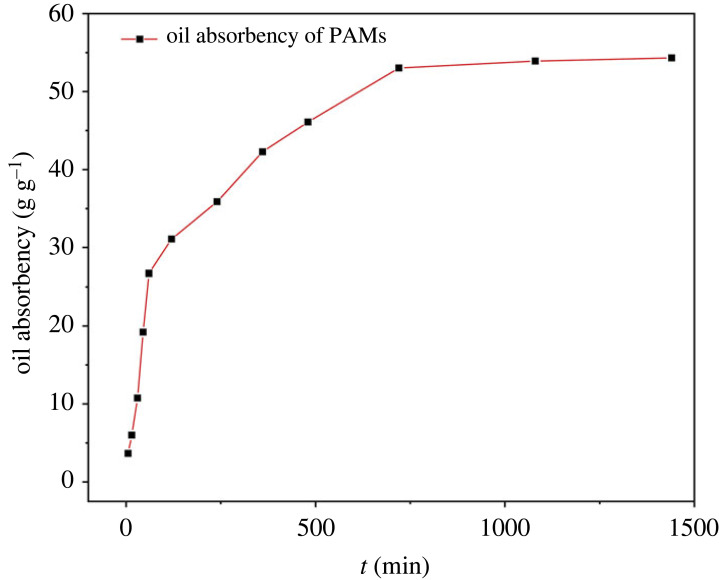
Time-dependent absorbency of PAMs for CHCl_3_.

The oil absorbency of PAMs as a function of absorption time is given in figure 9. Apparently, the oil absorbency of PAMs reached a maximum value after approximately 10 h in oils or organic solvents.

To preliminarily verify the absorption mechanism of PAMs, swelling kinetic models were fitted by evaluating the oil absorbency of PAMs as a function of time. Then, CHCl_3_ was chosen to study the oil-absorbing swelling kinetic process. The critical values of the first-order and second-order sorption kinetics are given in table 4.

**Table 4.** Linear fitting equation of the two swelling kinetic models.

swelling kinetics	first-order sorption kinetics	second-order sorption kinetics
linear fitting	*y* = 0.0017*x* + 0.0934	*y* = (2.4121 × 10^−4^)*x*−0.00204
*K*/(1/min)	*K*_1_ = 0.0039	*K*_2_ = 2.4121 × 10^−4^
*R*^2^	0.9546	0.9575

**Figure 10.**
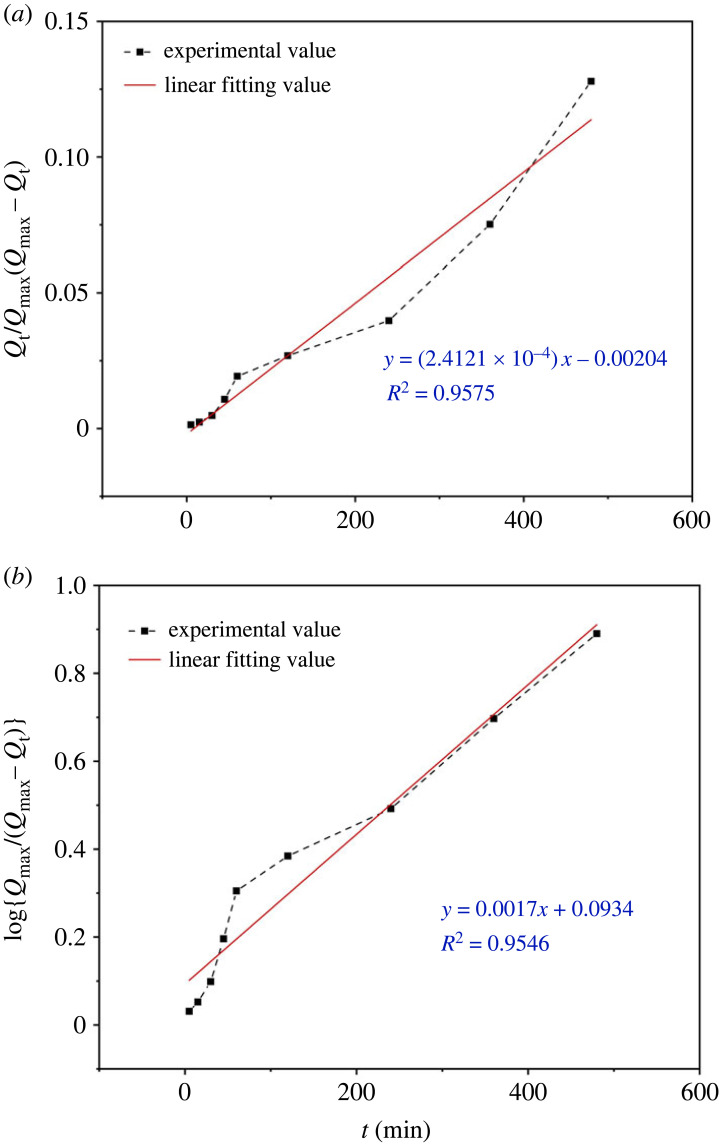
Kinetics models of the first-order sorption (*a*) and the second-order sorption (*b*) of PAMs.

The linear fitting curves of the two kinetics models are illustrated in figure 10. The *R*^2^ of the first-order sorption process showed a tiny gap with second-order sorption. Nonetheless, because of the limitation of the first-order sorption model, it was more suitable for describing the uncomplicated physical absorption or chemical absorption [41]. The sorption mechanism in the initial stage of PAMs was dominated by van der Waals forces and capillary dint, and the molecular diffusion dominated the sorption process [42,43]. This phenomenon might indicate that the second-order sorption kinetic was more suitable to describe a subsequent extensive swelling process in the oils of PAMs, which was dominated by chemical forces [44]. These processes were in accord with the sorption mechanism of oil-absorbing resin.

### 3.11. Reusability and oil retention of PAMs

**Figure 11.**
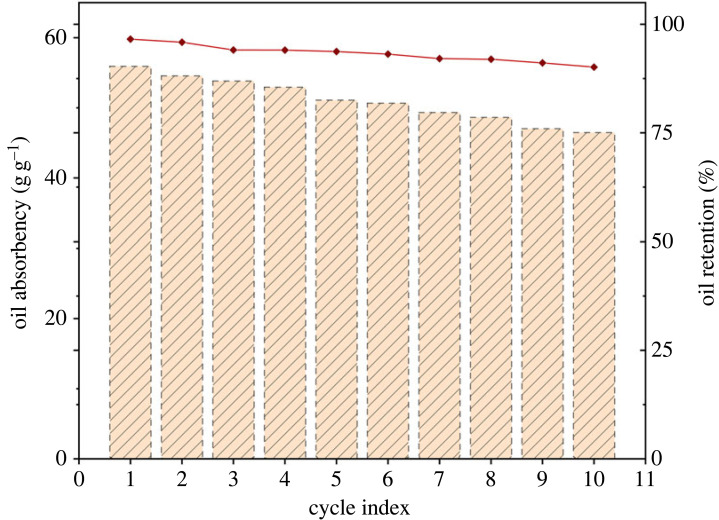
Reusability and oil retention of PAMs in CHCl_3_.

Reusability and oil retention are vital for the practical application of oil-absorbing resins [45]. To prove the above two abilities of PAMs, the PAMs were immersed in CHCl_3_ to evaluate their reusability and oil retention. The relevant results are shown in figure 11.

The oil absorbency of PAMs gradually declined after cycling but was still exhibit good oil absorbency for CHCl_3_. Simultaneously, the oil retention of PAMs was maintained above 90% within the range of 10 cycles. Accordingly, it was evidenced that the reusability and oil retention of PAMs had an insignificant fluctuation within at least 10 cycles in organic solvents.

The crosslinking degree of PAMs was 90.2%, and the crosslinking degree of PAMs after 10 cycles in organic solvents was 83.0%. These results indicate that oil-absorbing resin with MBA as a crosslinker synthesizes a good, effective crosslinked network. In addition, the most likely cause of the decrease in the crosslinking degree of the PAMs after 10 cycles in organic solvents was that part of the weaker crosslinked structure was destroyed during the processes of absorbing, desorbing and drying [39]. However, even after 10 cycles, PAMs still had a high crosslinking degree, which meets the requirements of repeated use.

### 3.12. Thermal and mechanical performance

**Figure 12.**
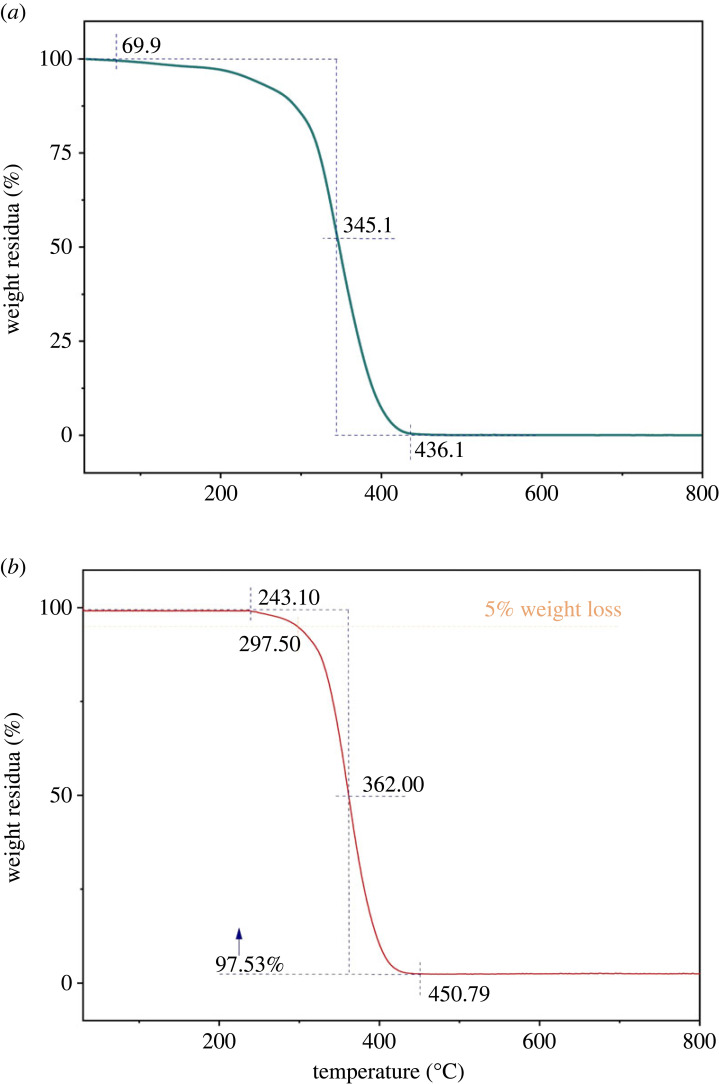
Thermogravimetric analysis (TGA) of the P(SMA-co-BA) (*a*) and PAMs (*b*) (heating rate: 10°C/min in a N_2_ atmosphere).

Thermal stability is described by the significant flame-retardant performance of oil-absorbing resin [46]. The weight loss of P(SMA-co-BA) and PAMs in the temperature range of 30–800°C is shown in figure 12*a*,*b*, respectively. PAMs exhibited a slight weight loss before 300°C in a N_2_ atmosphere, which indicates that PAMs had good thermal stability. However, P(SMA-co-BA) exhibited a greater weight loss before 300°C in a N_2_ atmosphere. Obviously, the thermal performance of the oil-absorbing resin was improved after introducing BZMA into the polymerization system. Specifically, P(SMA-co-BA) and PAMs started to decompose slowly at temperatures of 69.9 and 243.10°C, respectively, and finished the decomposition process at temperatures of 436.1 and 450.79°C, respectively, showing that PAMs could be used under normal absorptive conditions.

**Figure 13.**
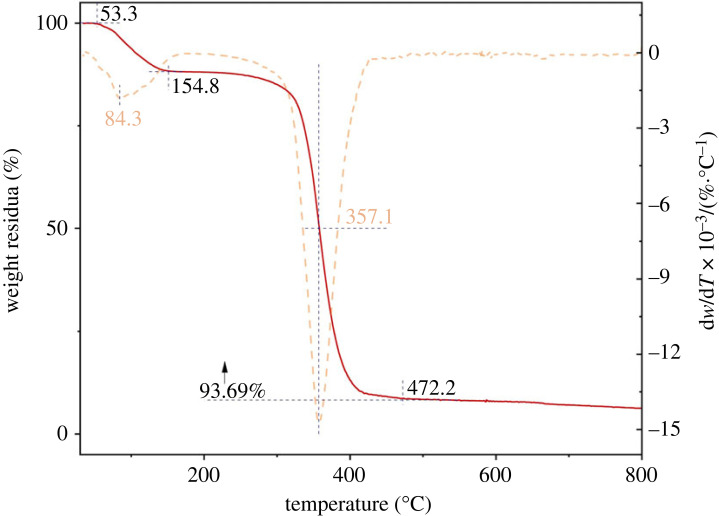
Thermogravimetric (TG) of PAMs absorbing oils In 10 cycles (heating rate: 10°C/min in a N_2_ atmosphere).

The TG and DTG curves in the temperature range of 30–800°C of PAMs that absorbed and desorbed oils repeatedly in 10 cycles are given in figure 13. The first peak of the DTG curve was attributed to the breaking of the chemical bonds that were weakened after cycle swelling and deswelling, and the degradation of oil that could not be extracted by ethanol. Evidently, a slight weight loss of PAMs occurred before 154.8°C in a N_2_ atmosphere, which demonstrates that repeated usage of PAMs had little effect on the thermal stability.

The elastic modulus and tensile strength of P(SMA-co-BA) are 0.0109 and 0.0286 MPa, respectively. The elastic modulus and tensile strength of PAMs are 0.0181 and 0.0306 MPa, respectively. Furthermore, the tensile testing results of P(SMA-co-BA) and PAMs are shown in electronic supplementary material, figure S3. It is evident that the addition of BZMA in the polymerization system enhanced the material's rigidity and toughness. The stress–strain tensile curves of P(SMA-co-BA) and PAMs are shown in electronic supplementary material, figure S3.

### 3.13. Oil-water separation performance of PAMs

**Figure 14.**
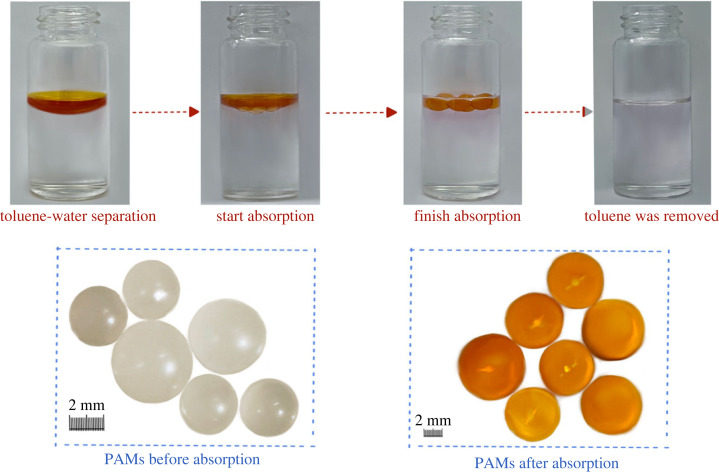
Oil absorption process of the PAMs in an oil-water mixture.

The water absorbency of PAMs was tested using the same method as for oil absorbency, and the results showed that the PAMs did not adsorb water at all. Then, CHCl_3_ was stained by oil-soluble methyl red and mixed with water, and the PAMs were placed into the oil-water mixture, as shown in figure 14.

The oil-absorbing resin dispersed on the oil-water boundary because of the density difference. When the resin was removed after oil absorption, changes in the colour and size of PAMs were observed. Simultaneously, it was observed that the stained organic solvent was entirely absorbed by PAMs.

These results showed that the PAMs soaked in organic solvent swelled to achieve oil absorption, which proves the potential of PAMs for oil-water separation.

## 4. Conclusion

Oil-absorbing resins (PAMs) were successfully synthesized by introducing commercially available BZMA as a functional monomer via an uncomplicated polymerization process. The FT-IR results confirmed the existence of benzene rings as functional groups in the internal network structure of PAMs. According to the test of the optimizing synthesis parameters, the best performance was observed when the ratio of the monomers was *ω* (SMA : BA : BZMA) = 1 : 0.2 : 0.2, the water and oil ratio was 5 : 1, and the concentrations of BPO, PVA and MBA were 0.5 *ω*t%, 0.5 *ω*t% and 0.5 *ω*t%, respectively. The PAMs showed good oil absorbency, oil retention and thermal stability after 10 cycles of reuse in the organic solvent. It is believed that PAMs could potentially be used in the treatment of oily wastewater. The results of these experiments can be used as a reference for designing a synthesis system of oil-absorbing resin.

## Data Availability

The basic experimental data of this experiment are presented in the form of Word, and this part of data is allowed to be copied, distributed, transmitted and adapted by anyone [47].
